# Pseudogene-Derived lncRNAs and Their miRNA Sponging Mechanism in Human Cancer

**DOI:** 10.3389/fcell.2020.00085

**Published:** 2020-02-28

**Authors:** Weiyang Lou, Bisha Ding, Peifen Fu

**Affiliations:** ^1^Department of Breast Surgery, The First Affiliated Hospital, College of Medicine, Zhejiang University, Hangzhou, China; ^2^Program of Innovative Cancer Therapeutics, Division of Hepatobiliary and Pancreatic Surgery, Department of Surgery, First Affiliated Hospital, College of Medicine, Zhejiang University, Hangzhou, China; ^3^Key Laboratory of Combined Multi-Organ Transplantation, Ministry of Public Health, Key Laboratory of Organ Transplantation, Zhejiang University, Hangzhou, China

**Keywords:** pseudogene, microRNA (miRNA), long non-coding RNA (lncRNA), circular RNA (circRNA), competing endogenous RNA (ceRNA), cancer

## Abstract

Pseudogenes, abundant in the human genome, are traditionally considered as non-functional “junk genes.” However, recent studies have revealed that pseudogenes act as key regulators at DNA, RNA or protein level in diverse human disorders (including cancer), among which pseudogene-derived long non-coding RNA (lncRNA) transcripts are extensively investigated and has been reported to be frequently dysregulated in various types of human cancer. Growing evidence demonstrates that pseudogene-derived lncRNAs play important roles in cancer initiation and progression by serving as competing endogenous RNAs (ceRNAs) through competitively binding to shared microRNAs (miRNAs), thus affecting both their cognate genes and unrelated genes. Herein, we retrospect those current findings about expression, functions and potential ceRNA mechanisms of pseudogene-derived lncRNAs in human cancer, which may provide us with some crucial clues in developing potential targets for cancer therapy in the future.

## Background

Cancer, a major killer of human health, constitutes an enormous burden on society in both more and less economically developed countries. According to GLOBOCAN statistics, about 14.1 million new cancer cases were diagnosed and 8.2 million cancer-associated deaths occurred in 2012 all over the world (Torre et al., 2015). Cancer is a complex disease. Multiple lines of evidence suggest that numerous risk elements, containing genetic and environmental factors, account for cancer initiation and development, among which the involvement of mis-regulation of non-coding RNAs (ncRNAs) in cancer has aroused extensive attention during the past few decades.

Based on length, ncRNAs can be divided into several subtypes, such as microRNA (miRNA), long non-coding RNA (lncRNA) as well as circular RNA (circRNA). ncRNAs, especially miRNA, have been solely studied until a famous hypothesis, namely competing endogenous RNA (ceRNA) mechanism, which was proposed by the team of Salmena et al. (2011). ceRNA hypothesis represents a hidden RNA language that messenger RNA (mRNA) can cross talk with lncRNA or circRNA by competitively binding to shared miRNAs, and eventually influence physiological and pathological processes (Ou et al., 2019; Wang W. et al., 2019; Zhang et al., 2020). After the emergence of ceRNA hypothesis, ncRNA-related research has been developed rapidly.

Jacq et al. (1977) introduced a new word “pseudogene” when they discovered a copy of the 5S rRNA gene in Xenopus laevis. The copy with 5′-end truncation and 14-bp mismatches makes it non-functional. From then on, a large number of pseudogenes have been gradually found in a variety of prokaryotes and eukaryotes, including homo species. For a long time, pseudogenes are considered as non-functional “junk genes,” “relics of evolution” or “genomic fossil.”

In recent years, with the huge advancement of in-deep research, multilayered functions of pseudogene DNAs, RNAs or proteins have been reported in various types of human cancer (Xiao-Jie et al., 2015), among which pseudogene-derived RNA transcripts are the most investigated. Pseudogene-expressed RNAs are key components of lncRNAs. Similar as common lncRNAs, pseudogene-derived lncRNAs can also function as critical modulators in initiation and development of human cancer by ceRNA mechanism *via* sponging miRNAs, which is supported by increasing findings. Therefore, in this work, we highlight recent findings regarding the expression, function and miRNA sponging mechanism of pseudogene-derived lncRNAs in diverse types of human cancer.

## Origination and Classification of Pseudogenes and Pseudogene-Derived lncRNAs

Based on the origination form from its ancestral gene, pseudogenes can be classified into three types: (1) processed pseudogenes or retrotransposed pseudogenes deriving from retrotransposition of processed mRNA back into the genome; (2) unprocessed pseudogenes or duplicated pseudogenes deriving from unfaithful gene duplications; and (3) unitary pseudogenes deriving from gene mutations (Xiao-Jie et al., 2015). lncRNAs are divided into several categories according to genomic organization and relation to coding genes, such as long intergenic non-coding RNAs, antisense RNAs, sense overlapping RNAs, sense intronic RNAs, enhancer RNAs as well as pseudogene-expressed lncRNAs (Grander and Johnsson, 2016). Although only few of pseudogenes can be transcribed, all the three types of pseudogenes may transcribe and are called transcribed pseudogenes or pseudogene-derived transcripts. However, compared with other members of lncRNAs, transcribed pseudogenes-derived lncRNAs have not been paid attention previously. Recent studies have demonstrated that transcribed pseudogene-derived lncRNAs play important roles in multiple biological processes, such as cell proliferation, cell cycle, cell migration and cell death (Lai et al., 2019; Oliveira-Mateos et al., 2019; Varesio et al., 2019).

## Competing Endogenous RNA Mechanism of Pseudogene-Derived lncRNA

Previous evidences have suggested that pseudogene-expressed RNAs could function as antisense RNAs or endo-siRNAs (Korneev et al., 1999; Watanabe et al., 2008). Besides, recent studies have also found that pseudogene-expressed RNAs serve as sponges of miRNAs and thus exert biological roles. To better understand the miRNA sponge mechanism of pseudogene-derived lncRNAs in cancer, competing endogenous RNA (ceRNA) mechanism proposed by Salmena et al. (2011) should be introduced. In this hypothesis, messenger RNA, lncRNA and circRNA can “talk” to each other by binding to shared miRNAs using miRNA response elements (MREs). Dysregulation of lncRNAs, pseudogenes and circRNAs leads to alteration of abundance of miRNAs, thus affecting their inhibition of downstream target expression. This mechanism also applies to pseudogene-derived transcripts. To date, ceRNA mechanism is validated to participate in initiation and progression of human cancer when its dysregulated (Qu et al., 2015; Yang C. et al., 2016). Based on ceRNA mechanism, researchers and scholars have discovered a variety of potential cancer-associated pseudogenes using *in silico* analysis. For example, Wei Y. et al. (2017) identified three pseudogene-involved ceRNA triples in lung adenocarcinoma, including NKAPP1-miR-21-5p-PRDM11, MSTO2P-miR-29c-3p-EZH2 and RPLP0P2-miR-29c-3p-EZH2; Jiang et al. (2018) screened several prostate cancer-related pseudogenes by establishing pseudogene-miRNA-gene triple ceRNA regulatory network. Lab experiments also confirmed the involvement of pseudogene-mediated ceRNA mechanism in cancer development. For instance, HMGA1 pseudogenes, HMGA1P6 and HMGA1P7, were reported to serve as candidate proto-oncogenic ceRNAs (Esposito et al., 2014); HMGA1P7 was also found to sustain overexpression of H19 and lgf2 by acting as decoy for miR-15, miR-16, miR-214, and miR-761 (De Martino et al., 2016). Karreth et al. (2015) suggested that BRAF pseudogenes BRAF-RS1 and BRAFP1 functioned as ceRNAs to elevate BRAF expression and activate MAPK signaling, thereby eliciting their roles in lymphoma.

## Expression and Functions of Pseudogene-Derived lncRNAs in Human Cancer

**FIGURE 1:**
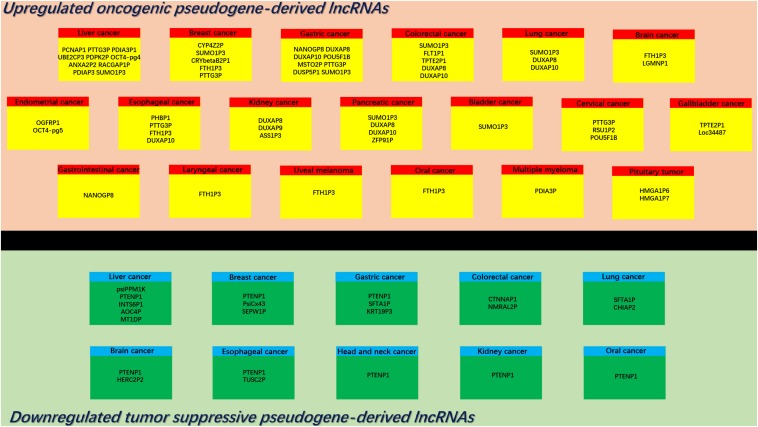
Overview of reported upregulated and downregulated pseudogene-derived lncRNAs in diverse types of human cancer.

Dysregulation of pseudogenes and their transcripts has been implicated into initiation and/or progression of human disorders, including cancer. Among pseudogene-derived lncRNAs, some act as tumor promotors, facilitating cancer development, whereas the other function as tumor suppressors, inhibiting cancer progression. In this part, we summarized the upregulated oncogenic pseudogene-derived lncRNAs and downregulated tumor suppressive pseudogene-derived lncRNAs in diverse types of human cancer (Figure 1).

## Upregulated Oncogenic Pseudogene-Derived lncRNAs

### Liver Cancer

As is known to all, hepatitis B virus (HBV) infection is closely linked to occurrence of hepatocellular carcinoma (HCC). PCNAP1 was reported to boost HBV replication, thus enhancing tumor growth of HCC (Feng et al., 2019). lncRNA PTTG3P was markedly upregulated in HCC samples compared with normal samples and its overexpression promoted growth and metastasis of HCC by increasing its cognate gene PTTG1 and activating PI3K/AKT signaling pathway (Huang J.L. et al., 2018). p53 signaling is a widely acknowledged tumor suppressive pathway in multiple human cancers, including HCC (Masutomi et al., 2001). PDIA3P1, upregulated in HCC tissues compared with paired normal adjacent tissues, could increase proliferation, migration and invasion, and decrease apoptosis of HCC by suppressing p53 signaling pathway (Kong et al., 2017). UBE2CP3 was significantly upregulated in HCC and was discovered to function as an oncogene by increasing tumor metastasis through inducing epithelial-mesenchymal transition (Cao et al., 2017). Lin et al. (2018) also confirmed the oncogenic role of UBE2CP3 in HCC, facilitating angiogenesis *via* activation of ERK1/2/HIF-1alpha/VEGFA signaling. Another highly expressed pseudogene-derived lncRNA, PDPK2P, also exhibited these oncogenic effects on HCC by PDK1/AKT/caspase 3 signaling pathway (Pan et al., 2019). OCT4-pg4, acting as a natural sponging of miR-145 to upregulating OCT4, exerted an oncogenic role in hepatocarcinogenesis by promoting growth (Wang et al., 2013). HCC is notorious for its high aggressiveness and easy to relapse. A recent study conducted by the team of Wang Q demonstrated that ANXA2P2 was highly expressed in HCC and promoted an aggressive phenotype of HCC (Wang Q.S. et al., 2019). And Wang M.Y. et al. (2019) confirmed that RACGAP1P facilitated early recurrence of HCC by activating RACGAP1/Rho/ERK signaling axis. Dysregulation of pseudogenes and their transcripts accounts for the development of chemo-resistance and radio-resistance of HCC which greatly reduces the efficacy of chemotherapy and radiotherapy. For example, Xie et al. (2019) suggested that PDIAP3 caused doxorubicin resistance of HCC by targeting miR-125/124-TRAF6/NF-KB signaling; Zhou Y. et al. (2019) found that knockdown of SUMO1P3 markedly enhanced radio-sensitivity in HCC.

### Breast Cancer

CYP4Z2P, an oncogenic pseudogene of CYP4Z1, has been widely investigated in breast cancer. The 3′UTR of CYP4Z2P was found to promote angiogenesis of breast cancer (Zheng et al., 2014, 2015). The ceRNA network of CYP4Z2P and its parental gene CYP4Z1 exerted an anti-apoptotic function in breast cancer (Li C. et al., 2017). Tamoxifen is an effective therapy for estrogen receptor (ER)-positive breast cancer. Zheng et al. (2016) discovered that the ceRNA network of CYP4Z2P and CYP4Z1 conferred tamoxifen resistance of breast cancer. The ceRNA network, activated by transcriptional factor six2, was also responsible for maintaining the stemness of breast cancer (Zheng L. et al., 2019). In addition to CYP4Z2P, some pseudogene-derived lncRNAs were also found to function as oncogenes in breast cancer. For example, Liu et al. (2017a) confirmed that SUMO1P3 promoted proliferation, migration and invasion of breast cancer; Barrow et al. (2019) found that CRYbetaB2P1 was upregulated in breast cancer and facilitated progression of breast cancer by increasing CRYbetaB2 expression; and Wang R. et al. (2018) demonstrated that FTH1P3 led to paclitaxel resistance of breast cancer. PTTG3P was markedly upregulated in breast cancer samples compared with normal controls and its upregulated linked to poor prognosis of patients with breast cancer (Lou et al., 2019).

### Gastric Cancer

NANOGP8 expression was significantly elevated in gastric cancer and knockdown of NANOGP8 resulted in decreased proliferation and promoted apoptosis of gastric cancer cells (Li et al., 2019). Ma X. et al. (2018) also suggested that NANOGP8 was a positive regulator of gastric cancer stem cells, associated with epithelial-mesenchymal transition, stemness, cancer stem cell markers as well as Wnt signaling pathway. DUXAP8, a 2107 nt RNA derived from pseudogene, was obviously upregulated in gastric cancer and significantly associated with greater tumor size, advanced clinical stage and lymphatic metastasis (Ma et al., 2017). Overexpression of DUXAP8 promoted cell proliferation and migration of gastric cancer by epigenetically inhibiting PLEKHO1 expression. Another DUXA-associated pseudogene, DUXAP10, also exerted its oncogenic roles in gastric cancer by transcriptionally repressing LATS1 expression and maintaining the stability of beta-catenin mRNA (Xu et al., 2018). POU5F1B, a processed pseudogene highly homologous to OCT4, was frequently amplified in gastric cancer cell lines and clinical specimens when compared with their corresponding controls (Hayashi et al., 2015). Furthermore, after POU5F1B overexpression in gastric cancer, a variety of growth factors were induced and an aggressive phenotype was exhibited. Ni et al. (2017) showed that MSTO2P was markedly associated with lymphatic metastasis and distal metastasis and promoted growth, colony formation, migration and invasion of gastric cancer. High expression PTTG3P linked to large tumor size, increased tumor invasiveness and served as an unfavorable prognostic biomarker (Weng, 2017). Besides, the abilities of gastric cancer proliferation and invasion were enhanced after ectopic expression of PTTG3P. Wang X. et al. (2019) found that DUSP5P1, directly induced by C8orf76, fueled gastric tumorigenicity and metastasis by activating MAPK signaling pathway. SUMO1P3 was significantly upregulated in gastric cancer and correlated with tumor size, differentiation, lymphatic metastasis and invasion (Mei et al., 2013). ROC curve analysis also indicated that high expression level of SUMO1P3 might be a potential diagnostic biomarker for gastric cancer. However, its functions in gastric cancer remain unclear and need to be further investigated.

### Colorectal Cancer

High SUMO1P3 level was reported to be associated with advanced histological stages, metastasis, angiogenesis and poor prognosis of colon cancer patients (Zhang L.M. et al., 2017). Inhibition of SUMO1P3 repressed proliferation, migration, invasion and pro-angiogenesis of colon cancer cells *in vitro*, and reduced growth, liver metastasis and vascularization of colon cancer *in vivo* by decreasing cyclin D1, vimentin and VEGFA but increasing *E*-cadherin expression. FLT1P1markedly promoted cell proliferation *in vitro* and xenograft tumor growth *in vivo* (Ye et al., 2015). TPTE2P1 levels in colorectal cancer tissues were higher than that in adjacent normal tissues, and its upregulation was markedly associated with poor patients’ survival (Dai et al., 2019). The authors also indicated that silencing expression of TPTE2P1 resulted in cell cycle arrest at S phase and caused cell apoptosis in colorectal cancer. Moreover, the tumor suppressive effects of TPTE2P1 on colorectal cancer was observed in the *in vivo* experiment. DUXAP8 was remarkably overexpressed in colorectal cancer and DUXAP8 knockdown led to inhibited proliferation, migration and invasion and enhanced apoptosis (Du et al., 2019). Lian et al. (2017) suggested that another DUXA-associated pseudogene-derived lncRNA, DUXAP10, was also evidently upregulated in colorectal cancer and positively correlated with advanced pathological stages, larger tumor sizes and lymph node metastasis. DUXAP10 promoted cell proliferation and cell cycle progression and blocked cell apoptosis by epigenetically silencing expression of p21 and PTEN.

### Lung Cancer

SUMO1P3 expression was markedly increased in non-small cell lung cancer tissues and cells compared with corresponding normal controls and in metastatic lymph node specimens in comparison to primary tumor tissue specimens (Zhang et al., 2019). The authors also confirmed high SUMO1P3 expression was correlated with late clinical stage, lymph node metastasis, distant metastasis and poor differentiated degree. Moreover, SUMO1P3 enhanced cell migration and invasion of non-small cell lung cancer. Sun et al. (2017) reported that DUXAP8, upregulated in non-small cell lung cancer and an unfavorable prognostic biomarker, significantly facilitated cell growth, migration and invasion, and impaired apoptosis both *in vitro* and *in vivo* by epigenetically silencing EGR1 and RHOB. DUXAP10 expression was identified to be overexpressed in non-small cell lung cancer tissues and cell lines and its upregulation was correlated with poor prognosis of patients with non-small cell lung cancer (Wei C.C. et al., 2017). Functional and mechanistic experiments suggested that DUXAP10 exerted oncogenic roles in non-small cell lung cancer by binding to LSD1 and epigenetic silencing expression of LATS2 and RRAD.

### Brain Cancer

Glioma and glioblastoma are two main types of brain cancer. Pseudogene-derived lncRNAs are found to facilitate development and progression of glioma and glioblastoma. For example, Zhang et al. (2018) FTH1P3 was reported to be upregulated in glioma tissues and high-grade glioma tissues when compared with normal brain tissues and low-grade glioma tissues, respectively. Overexpression of FTH1P3 enhanced glioma cell proliferation and inhibited apoptosis by regulating miR-224-5p/TPD52 pathway. Another study by Xu et al. (2019) demonstrated that LGMNP1 was significantly increased in glioblastoma cells after radiation and its overexpression conferred radio-therapy resistance in glioblastoma by reducing DNA damage and apoptotic population.

### Endometrial Cancer

The expression level of OGFRP1 was significantly upregulated in endometrial cancer (Lv et al., 2019). Lv et al. (2019) also experimentally confirmed that knockdown of OGFRP1 suppressed the malignant behaviors of endometrial cancer, including suppressed cell viability, enhanced cell apoptosis and inhibited cell migration and invasion. OCT4-pg5 was found to be aberrantly activated in endometrial cancer samples compared with benign endometrium samples, and increased expression of OCT4-pg5 enhanced proliferation by promoting OCT4/PI3K/AKT/CCND1 signaling (Bai et al., 2015).

### Esophageal Squamous Cell Carcinoma

Feng et al. (2017) reported that PHBP1, markedly upregulated in esophageal squamous cell carcinoma and positively correlated with clinical advanced stage, facilitated proliferation, colony formation and xenograft tumor growth *in vitro* and *in vivo* by inducing cell cycle arrest. PTTG3P expression was found to increase in esophageal squamous cell carcinoma tissues and cell lines (Zhang and Shi, 2019). Enhanced expression of PTTG3P greatly stimulated migration and invasion of esophageal squamous cell carcinoma through upregulating expression levels of PTTG1 and PTTG2 (Zhang and Shi, 2019). Yang L. et al. (2018) demonstrated that FTH1P3 was notably upregulated in esophageal squamous cell carcinoma and knockdown of FTH1P3 significantly inhibited proliferation, migration and invasion of esophageal squamous cell carcinoma cells by regulating SP1/NF-kB signaling. The study conducted Wang Z. et al. (2018) certified an obvious increased expression of DUXAP10 in esophageal squamous cell carcinoma. They also showed that DUXAP10 was positively correlated with poor prognosis and epigenetically silenced p21 by recruiting EZH2 to the promoter of p21, thereby promoting cell proliferation and metastasis.

### Renal Cell Carcinoma

Chen et al. (2019) demonstrated that DUXAP8 and DUXAP9 were significantly upregulated in renal cell carcinoma, promoted tumor growth and served as two unfavorable prognostic biomarkers for patients with renal cell carcinoma. DUXAP8 was also discovered to enhance progression of renal cell carcinoma (Huang T. et al., 2018). Androgen receptor plays key roles in development of renal cell carcinoma. Wang K. et al. (2019) found androgen receptor could decrease the expression of ASS1P3, a pseudogene of ASS1, and thus facilitate cell growth of renal cell carcinoma.

### Pancreatic Cancer

The oncogenic function of SUMO1P3 in the development of pancreatic cancer was reported by Tian et al. (2018). They found that SUMO1P3 expression was markedly elevated in pancreatic cancer tissues when compared with normal controls. Functional experiments revealed the enhanced effect of SUMO1P3 on proliferation, migration and invasion of pancreatic cancer. By epigenetically silencing CDKN1A an KLF2, DUXAP8, upregulated in pancreatic cancer, promoted growth of pancreatic cancer (Lian et al., 2018b). The team of Lian et al. (2018a) demonstrated that DUXAP10 expression was higher in renal cell carcinoma patients with an advanced TNM stage and positive lymph node metastasis. They also confirmed that knockdown of DUXAP10 could result in inhibited proliferation, migration, invasion and enhanced apoptosis in renal cell carcinoma through interacting with RNA-binding proteins, EZH2 and LSD1. Depletion of ZFP91P significantly decreased cell proliferation and migration capacities of pancreatic cancer by altering beta-catenin and vimentin expression (Huang et al., 2016).

### Other Types of Cancer

SUMO1P3 was increased in bladder cancer and its high expression predicted poor prognosis of patients with bladder cancer (Zhan et al., 2016). Furthermore, after knockdown of SUMO1P3, bladder cancer exhibited cell proliferation and migration inhibition and apoptosis induction (Zhan et al., 2016).

PTTG3P, a pseudogene of PTTG1 which is upregulated in many types of cancer, was found to promote cervical cancer growth and metastasis by enhancing PTTG1 expression (Guo et al., 2019). Recently, Liu et al. (2017c) showed that RSU1P2 was increased in cervical cancer and boosted the malignant phenotype of cervical cancer. POU5F1B was also reported to be upregulated in cervical cancer tissues and cell lines by Yu et al. (2019). The authors confirmed that interference with expression of POU5F1B significantly suppressed cell proliferation, migration and invasion, and induced apoptosis in cervical cancer (Yu et al., 2019).

Gallbladder is a rare malignant tumor with poor prognosis all over the world. Two pseudogene-derived lncRNAs, TPTE2P1 and Loc344887, have been documented to play oncogenic roles in development of gallbladder cancer (Lv et al., 2015; Wu et al., 2017). Depletion of TPTE2P1 significantly blocked epithelial-mesenchymal transition, migration and invasion of gallbladder cancer (Lv et al., 2015). Loc344887 was elevated in gallbladder cancer, was positively associated with larger tumor size, and facilitated cell proliferation, cell cycle progression, migration and invasion (Wu et al., 2017).

Uchino et al. (2012) suggested that NANOGP8 overexpression remarkably increased cell proliferation whereas its inhibition suppressed the proliferation in human gastrointestinal cancer cells.

Yuan et al. (2019) confirmed FTH1P3 was significantly upregulated in laryngeal squamous cell cancer and positively linked to the poor differentiation, high T classification, positive lymph node metastasis and advanced clinical stage. The authors also demonstrated that FTH1P3 overexpression resulted in enhancement of cell proliferation, migration and invasion and inhibition of cell apoptosis in laryngeal squamous cell cancer (Yuan et al., 2019).

FTH1P3 expression was also increased in uveal melanoma and elevated expression of FTH1P3 promoted cell proliferation, cell cycle and migration of uveal melanoma (Azzariti et al., 2017).

The group of Zang (2017) suggested that FTH1P3 facilitated cell proliferation and colony formation in oral squamous cell carcinoma by upregulating the expression of fizzled 5.

Yang X. et al. (2018) demonstrated that PDIA3P was highly expressed in multiple myeloma, was correlated with poor prognosis of patients with multiple myeloma, and induced growth and drug resistance of multiple myeloma.

Two pseudogene-derived lncRNAs, HMGA1P6 and HMGA1P7, contributed to increase its cognate gene HMGA1 level in human pituitary tumor and then to pituitary tumorigenesis (Esposito et al., 2015).

## Downregulated Tumor Suppressive Pseudogene-Derived lncRNAs

### Liver Cancer

psiPPM1K, a retrotranscript pseudogene, was significantly reduced in HCC surgical specimens (Chan et al., 2013). Two endo-siRNAs originated from psiPPM1K are found to inhibit oncogenic cell growth of HCC. Low expression of lncRNA PTENP1 in HCC has been reported (Chen et al., 2015). A similar result was also identified by another study conducted by Qian et al. (2017). They found that PTENP1 suppress HCC migration and invasion through enhancing PTEN signaling by interacting with miR-193a-3p. INTS6P1 upregulated its cognate gene INTS6 through competitively binding to miR-17-5p, thereby functioning its tumor suppressive roles in HCC (Peng et al., 2015). Wang et al. (2015) indicated that AOC4P inhibited metastasis of HCC by enhancing degradation of vimentin and suppressing epithelial-mesenchymal transition. MT1DP, directly inhibited by YAP and Runx2, resulted in reduced cell proliferation and colony formation in soft agar, but increased apoptosis in liver cancers (Yu W. et al., 2014).

### Breast Cancer

PTENP1 was reported as a tumor suppressor in development and progression of breast cancer. For instance, a recent study conducted by Tang et al. (2017a) showed that PTENP1 suppressed proliferation and migration of breast cancer by inhibiting AKT and MAPK signaling pathways; another study performed by Gao et al. (2019) demonstrated that PETNP1 increased expression of PTEN, thus mediating proliferation, invasion and drug resistance of breast cancer by activation of PI3K/AKT pathway. Connnexin 43 pseudogene, PsiCx43, increased sensitivity to cytotoxic chemotherapy of breast cancer (Bier et al., 2009). SEPW1P RNA was also validated to participate in PIWI-interacting RNA-36712-mediating suppression of breast cancer progression (Tan et al., 2019).

### Gastric Cancer

PTENP1 has been demonstrated to function as a tumor suppressor in several cancer cells, containing gastric cancer. For example, Guo et al. (2016) found that PTENP1 was frequently downregulated in gastric cancer, and suppressed proliferation, migration, invasion and promoted apoptosis of gastric cancer through increasing PTEN expression; Zhang R. et al. (2017) also confirmed the tumor suppressive roles of PTENP1 in gastric cancer by decoying miR-106b and miR-93 and thus upregulating PTEN expression. Apart from PTENP1, some pseudogene-derived lncRNAs were also demonstrated to be downregulated in gastric cancer and play tumor suppressive roles in gastric cancer progression. SFTA1P, which is 693 nt long, was obviously decreased in gastric cancer tissues compared with the adjacent normal tissues (Ma H. et al., 2018). Increased expression of SFTA1P suppressed proliferation, migration and invasion of gastric cancer by partially inhibiting p53 signaling. Downregulation of KRT19P3 in gastric cancer cells and tissues was confirmed by Zheng J. et al. (2019). Functional experiments suggested that enforced expression of KRT19P3 significantly blocked cell proliferation and metastasis both *in vitro* and *in vivo*. KRT19P3-mediated enhancement of COPS7A protein stability accounted for its roles in suppressing gastric cancer.

### Colorectal Cancer

Increasing evidence have supported the involvement of pseudogene-derived lncRNAs in the suppression of pathogenesis of cancer, including colorectal cancer. For example, Chen et al. (2016) confirmed that CTNNAP1 was significantly downregulated in colorectal cancer. The degree of CTNNAP1 dysregulation was markedly correlated with TNM stage. Furthermore, overexpression of CTNNAP1 could suppressed cell proliferation and tumor growth *in vitro* and *in vivo via* induction of G0/G1 cell cycle arrest. Sulforaphane, an anticancer agent, exerts its effects partially by inducing Nrf2-dependent signaling. The investigation performed by the group of Johnson G demonstrated that silencing of NMRAL2P could protect against sulforaphane-mediated inhibition of cell growth, colony formation and migration in colon cancer (Johnson et al., 2017).

### Lung Cancer

By combination of bioinformatics analysis and experimental validation, Zhang H. et al. (2017) suggested that SFTA1P was downregulated in both lung adenocarcinoma and lung squamous cell carcinoma and inhibited tumor growth, migration and invasion. The downregulation of SFTA1P in lung squamous cell carcinoma was also reported by the team of Li L. et al. (2017). They found elevated SFTA1P induced apoptosis and enhanced the sensitivity to cisplatin of lung squamous cell carcinoma. Intriguingly, low expression of SFTA1P was only correlated with lung adenocarcinoma patients’ poor prognosis but not with lung squamous cell carcinoma patients’ prognosis (Zhang H. et al., 2017). Besides, Shang et al. (2019) confirmed that CHIAP2 expression was markedly decreased in lung adenocarcinoma. After upregulation of CHIAP2, the impaired proliferation and invasion of lung adenocarcinoma were observed.

### Brain Cancer

PTENP1 is a tumor suppressive pseudogene-derived lncRNA reported in multiple types of cancer as well as brain glioma. Hu et al. (2019) reported that expression of PTENP1 was decreased in glioma tissues compared with normal brain tissues and forced expression of PTENP1 inhibited cell proliferation and migration *in vitro* by inducing p21 expression and blocking p38 signaling pathway. In addition, Yang C. et al. (2019) first identified HERC2P2 as a potential tumor suppressor in glioma by constructing a ceRNA network based on the Chinese Glioma Genome Atlas database. Subsequently, they also validated that HERC2P2 overexpression attenuated migration and colony formation abilities of glioma *in vitro* and inhibited glioma xenograft growth *in vivo*.

### Esophageal Squamous Cell Carcinoma

The tumor suppressive role of PTENP1 in esophageal squamous cell carcinoma was reported by Gong et al. (2017). They found a downregulated expression of PTENP1 in esophageal squamous cell carcinoma compared with the corresponding adjacent normal tissues and overexpression of PTENP1 inhibited tumor proliferation (esophageal squamous cell carcinoma). Liu et al. (2018) suggested TUSC2P was decreased in esophageal squamous cell carcinoma and its downregulation linked to unfavorable prognosis of patients with esophageal squamous cell carcinoma. The inhibitory effects of TUSC2P on proliferation and invasion of esophageal squamous cell carcinoma were also experimentally validated in the study (Liu et al., 2018). TUSC2P-mediated upregulation of TUSC2 was partially responsible for the tumor suppressive roles of TUSC2P in esophageal squamous cell carcinoma (Liu et al., 2018).

### Other Types of Cancer

PTENP1 was downregulated in head and neck squamous cell carcinoma and its low expression correlated with poor patients’ prognosis (Liu et al., 2017b). Upregulation of PTENP1 led to inhibited proliferation, colony formation and migration *in vitro* and suppressed growth *in vivo* in head and neck squamous cell carcinoma (Liu et al., 2017b).

PTENP1 was reported to act as a tumor suppressor in clear-cell renal cell carcinoma by Yu G. et al. (2014). They found that PTENP1 was downregulated in clear-cell renal cell carcinoma and overexpression of PTENP1 reduced cell proliferation, migration, invasion *in vitro* and tumor growth and metastasis *in vivo* (Yu G. et al., 2014).

Gao et al. (2017) suggested that PTENP1, protecting PTEN transcripts from being inhibited by miR-21, suppressed proliferation and colony formation of oral squamous cell carcinoma.

## ceRNA Mechanism Contributes to the Roles of Pseudogene-Derived lncRNAs in Human Cancer

Over the past decades, pseudogenes have been documented to play crucial roles in diverse cancer types in DNA, RNA and protein levels (Panagopoulos et al., 2008; Xiao-Jie et al., 2015). Considering study progresses, this work mainly reviewed the miRNA sponging function of pseudogene-derived lncRNAs in human cancer (Table 1).

**TABLE 1:** Summary of dysregulated pseudogenes and its molecular mechanism in diverse types of cancer.

**Name**	**Cancer type**	**Function**	**Binding miRNAs**	**Pathway**	**References**
WTAPP1	Multiple cancer	Promote migration and angiogenesis of endothelial progenitor cells	miR-3120	Akt/PI3K	Watanabe et al., 2008
FLT1P1	Colorectal cancer	Promote proliferation and growth	miR-520a	VEGFR1, VEGF-A	Ye et al., 2015
TUSC2P	Esophageal squamous cell carcinoma	Inhibit proliferation and invasion and promote apoptosis	miR-17-5p/miR-520a-3p/miR-608/miR-661		Liu et al., 2018
	Multiple cancer	Inhibit proliferation, survival, migration, invasion and colony formation and increases cell death	miR-17/miR-93/miR-299-3p/miR-520a/miR-608/miR-661		Korneev et al., 1999
SUM01P3	Non-small cell lung cancer	Promote cell migration and invasion	miR-136		Zhang et al., 2019
SEPW1P	Breast cancer	Promote cell proliferation, invasion and migration and confer chemoresistance	miR-7/miR-324	p53 signaling	Tan et al., 2019
RSU1P2	Cervical cancer	Promote tumor role	let-7a	IGF1R, N-myc and EphA4	Liu et al., 2017c
RP11-564D11.3	Hepatocellular carcinoma	Correlate with poor overall survival of patients	miR-200b-3p/miR-200a-3p/miR-429/miR-101-3p/miR-9-5p/miR-200c-3p/miR-141-3p	VEGFA	Watanabe et al., 2008
RP11-424C20.2	Hepatocellular carcinoma and thymoma	Correlate with immune infiltration	miR-378a-3p	UHRF1, IFN-gamma-mediated CLTA-4 and PD-L1 pathway	Watanabe et al., 2008
RACGAP1P	Hepatocellular carcinoma	Promote cell growth and migration	miR-15-5p	RACGAP1/Rho/ERK signaling axis	Watanabe et al., 2008
PTTG3P	Breast Cancer	Correlate with poor prognosis	miR-129-5p/miR-376c-3p/miR-383-5p		Lou et al., 2019
	Hepatocellular carcinoma	Promote cell proliferation, migration and invasion, and suppress cell apoptosis	miR-383	CCND1/PARP2 axis and PI3K/Akt signaling	Korneev et al., 1999
PTNEP1	Breast Cancer	Inhibit tumor progression	miR-26a	PTEN, AKT/mTOR signaling	Asselta et al., 2018
	Hepatocellular carcinoma	Inhibit cell proliferation, migration, invasion as well as induce autophagy and apoptosis	miR-17/miR-19b/miR-20a	PI3K/AKT pathway	Chen et al., 2015
	Oral squamous cell carcinoma	Inhibit proliferation	miR-21	PTEN	Gao et al., 2017
	Esophageal squamous cell	Inhibit growth and correlate with prognosis	miR-17-5p	SOCS6	Gong et al., 2017
	Breast cancer	Suppress cell proliferation, migration and invasion and promote cell apoptosis	miR-19b	p53 signaling, p-AKT	Li R.K. et al., 2017
	Breast Cancer	Inhibits cell proliferation and migration	miR-19b	PI3k/Akt pathway	Shi et al., 2018
	Multiple cancer	Glycogen synthesis	miR-499-5p	Insulin signaling	Wang et al., 2016
	Clear-cell renal cell carcinoma	Suppress cancer progression	miR-21		Yu G. et al., 2014
	Gastric cancer	Tumor suppressive role	miR-106b/miR-93		Zhang R. et al., 2017
	Bladder cancer	Suppress cancer progression	miR-17		Zheng et al., 2018
PDIA3P1	Hepatocellular carcinoma	Confer chemoresistance	miR-125a/miR-125b/miR-124	TRAF6 and NF-kB signaling	Xie et al., 2019
OCT4-pg5	Endometrial carcinoma	Promote proliferation	miR-145	OCT4/PI3K/AKT/cyclin D1 signaling	Bai et al., 2015
OCT4-pg4	Hepatocellular carcinoma	Promote growth and tumorigenicity	miR-145	OCT4	Wang et al., 2013
MSTO2P1	Gastric cancer	Promote the proliferation and colony formation	miR-335		Li R.K. et al., 2017
INTS6P1	Hepatocellular carcinoma	Suppress cancer progression	miR-17-5p	INTS6	Peng et al., 2015
HMGA1P6 and HMGA1P7	Breast cancer	Promote cancer progression	miR-16	H19 and IGF2	De Martino et al., 2016
	Multiple cancer	Correlate with advanced cancer stage and poor prognosis	miR-483/miR-675	Egr1	De Martino and Palma, 2017
	Thyroid carcinoma	Promote tumor role	miR-15/miR-16/miR-214/miR-761	HMGA1	Esposito et al., 2014
FTHIP3	Uveal melanoma	Promote uveal melanoma cell proliferation, cell cycle and migration.	miR-224-5p		Azzariti et al., 2017
	Prostate cancer	Promote prostate cancer progression	miR-638		Chan et al., 2018
	Breast cancer	Increase the 50% inhibitory concentration value of paclitaxel and block cell cycle arrest at G2/M phase	miR-206	ABCB1	Wang Z. et al., 2018a
	Oral squamous cell carcinoma	Facilitate cell proliferation and colony formation	miR-224-5p	Fizzled 5	Zhang, 2017
	Glioma	Promote glioma cell proliferation and inhibited apoptosis.	miR-224-5p	TPD52	Zhang et al., 2018
FOXO3P	Breast cancer	Suppress tumor growth and cancer cell proliferation and survival.	miR-22/miR-136*/miR-138/miR-149*/miR-433/miR-762/miR-3614-5p/miR-3622b-5p	FOXO3	Yang W. et al., 2016
DUXAP8 and DUXAP9	Renal cell carcinoma	promote growth of renal cell carcinoma	miR-29c-3p	COL1A1, COL1A2	Chen et al., 2019
	Colorectal cancer	Promote cell proliferation, migration and invasion, and suppress apoptosis	miR-577	STAT3/DUXAP8/miR-577/RAB14	Du et al., 2019
	Renal Cell Carcinoma	Enhances Renal Cell Carcinoma Progression	miR-126		Huang T. et al., 2018
CYP4Z2P	Breast cancer	Inhibit cell apoptosis	miR−125a−3p	AKT and ERK signaling pathways	Li C. et al., 2017
	Breast cancer	Promote angiogenesis	miR-211/miR-125a-3p/miR-197/miR-1226/miR-204	Phosphorylation of ERK1/2 and PI3K/Akt	Zheng et al., 2015
	Breast cancer	Render tamoxifen resistance	miR-125a-3p	CDK3-depended ERalpha activity	Zheng et al., 2016
CYP2A7P	Liver cancer		miR-126	CYP2A6	Nakano et al., 2015
CHIAP2	Lung adenocarcinoma	Inhibit proliferation and invasion	miR-3614-5p/miR-873-3p	Wnt pathway	Shang et al., 2019
ASSIP3	Renal cell carcinoma	Promote cell proliferation	miR-34a-5p	ASS1P3/miR-34a-5p/ASS1 signaling	Wang K. et al., 2019

### PTENP1

PTENP1 functions as a ceRNA and suppress tumor progression in multi-cancers, including breast cancer, HCC, oral squamous cell carcinoma, esophageal squamous cell carcinoma, clear-cell renal cell carcinoma, gastric cancer and bladder cancer. In breast cancer, PTENP1 was found to stimulates expression of PTEN transcript and inhibits cell proliferation and migration through decoying miR-19b (Li R.K. et al., 2017; Shi et al., 2018). Further, leads to p53 upregulation and p-AKT downregulation. In HCC, long non-coding RNA PTENP1 *via* its ceRNA interaction with miR-17, miR-19b and miR-20a, and finally suppressed cell growth through inhibited oncogenic PI3K/AKT pathway (Chen et al., 2015). Additionally, PTENP1 could mediated miR-21 expression and decreased the proliferation ability of oral squamous cell carcinoma and clear-cell renal cell carcinoma (Yu G. et al., 2014; Gao et al., 2017). Meanwhile, PTENP1 could suppress bladder cancer progression by regulating miR-17 as well as esophageal squamous cell carcinoma (Gong et al., 2017; Zheng et al., 2018). What’s more, PTENP1 functions to modulate PTEN expression by sponging miR-93 and miR-106b, plays a tumor suppressive role in gastric cancer (Zhang R. et al., 2017). Not only in cancer, PTEN also contributing to insulin resistance through binding miR-499-5p directly (Wang et al., 2016).

### FTH1P3

Increasing evidence has demonstrated that FTH1P3 functions as a ceRNA and participates in tumor initiation and development. For example, FTH1P3 facilitated progression of glioma (Zhang et al., 2018), uveal melanoma (Azzariti et al., 2017), and oral squamous cell carcinoma (Zhang, 2017). Furthermore, FTH1P3 induced paclitaxel resistance by regulating miR-206/ABCB1 axis in breast cancer (Wang R. et al., 2018). Chan et al. (2018) also suggested a FTH1 gene: pseudogene: microRNA modulated tumorigenesis in prostate cancer.

### FLT1P1

FLT1 encodes a member of the vascular endothelial growth factor receptor family. FLT1P1 is a bidirectionally transcribed pseudogene of FLT1. Ye et al. (2015) confirmed that FLT1P1 antisense transcript suppressed VEGFA by interaction with miR-520a and inhibited tumor cell proliferation and xenograft tumor growth.

### CYP4Z2P

CYP4Z2P and CYP4Z1 promoted angiogenesis of breast cancer by commonly targeting miR-211, miR-197, miR-1226, miR-125a, and miR-204 (Zheng et al., 2015). Moreover, the ceRNA network between CYP4Z1 and CYP4Z2P led to progression of breast cancer, including suppressed apoptosis (Li C. et al., 2017) and induced tamoxifen resistance (Zheng et al., 2016).

### DUXAP8 and DUXAP9

To date, two DUXA-associated pseudogene-derived lncRNAs, DUXAP8 and DUXAP9, have been discovered to act as ceRNAs to enhance cancer development. For example, Chen et al. (2019) demonstrated that DUXAP8 and DUXAP9 enhanced growth of renal cell carcinoma by binding to miR-29c-3p and leading to upregulation of COL1A1 and COL1A2; Huang T. et al. (2018) suggested that DUXAP8 facilitated progression of renal cell carcinoma by sponging miR-126. DUXAP8, upregulated by STAT3, also fueled migration and invasion of colorectal cancer by functioning as a ceRNA for miR-577 to regulate RAB14 (Du et al., 2019).

### HMGA1P6 and HMGA1P7

Esposito et al. (2014) for the first time, reported HMGA1 pseudogenes as candidate proto-oncogenic ceRNAs, including HMGA1P6 and HMGA1P7. HMGA1P7 was found to increase H19 and lgf2 expression by acting as decoy for miR-15, miR-16, miR-214, and miR-761 (De Martino et al., 2016). De Martino and Palma (2017) also showed that HMGA1P7 upregulated miR-483 and miR-675 through a ceRNA mechanism with Egr1.

### OCT4-pg4 and OCT4-pg5

Two OCT4-associated pseudogene-derived lncRNAs, OCT4-pg4 and OCT4-pg5, have been reported to act as ceRNAs, thus involving in cancer initiation and progression (Wang et al., 2013; Bai et al., 2015). OCT4-pg4 promoted growth and tumorigenicity of HCC by growing OCT4 expression through competing for miR-145 (Wang et al., 2013). OCT4-pg5 also upregulated OCT4 by sponging miR-145 and thus facilitated cell proliferation of endometrial carcinoma (Bai et al., 2015).

### PTTG3P

Pseudogene PTTG3P was found to be closely related with poor prognosis in HCC and breast cancer. Lou et al. (2019) suggested that PTTG3P expression positively associated with PTTG1 expression and may function by sponging miR-129-5p, miR-383-5p, and miR-376c-3p. Recently, Zhou Q. et al. (2019) reported that downregulation of PTTG3P promoted apoptosis and decreased proliferation, invasion and migration of HCC cells *via* increasing the expression levels of miR-383 targets, PARP2 and CCND1.

### TUSC2P

Liu et al. (2018) indicated that TUSC2P could suppress proliferation, invasion and accelerate apoptosis of esophageal squamous cell carcinoma *in vivo* and *in vitro* through modulating expression of miR-17-5p, miR-520a-3p, miR-608, and miR-661. Besides, it also could serve as a prognostic factor for patients with esophageal squamous cell carcinoma (Liu et al., 2018). In addition, TUSC2P was founded to act as a key tumor-inhibitor that could inhibits cell proliferation, migration and invasion in 4T1 and MDA-MB-231 cells (Rutnam et al., 2014). What’s more, TUSC2P promotes those functions by binding miR-17, miR-93, miR-299-3p, miR-520a, miR-608, and miR-661 according to the research conducted by Rutnam et al. (2014).

### ASS1P3

ASS1P3, a pseudogene of ASS1, promoted cell proliferation by functioning as a miRNA decoy for miR-34a-5p in renal cell carcinoma reported by the team of Wang K. et al. (2019).

### CHIAP2

Pseudogene-derived lncRNA, CHIAP2, suppressed lung adenocarcinoma cell proliferation and invasion by modulation of miR-3614-5p and miR-873-3p-mediated inhibition of NFATC2 and GSK3B (Shang et al., 2019).

### CYP2A7P

CYP2A7P was discovered to affect expression of its cognate gene CYP2A6 by functioning as a decoy for miR-126 in human liver (Nakano et al., 2015). However, the effect of CYP2A7P/miR-126/CYP2A6 axis in human cancer containing liver cancer is still not determined.

### FOXO3P

FOXO3P inhibited tumor growth and angiogenesis by activating FOXO3 activity (Yang W. et al., 2016). By luciferase reporter assay, the authors confirmed that FOXO3P exerted its roles through sponging several miRNAs, including miR-22, miR-136^∗^, miR-138, miR-149^∗^, miR-433, miR-762, miR-3614-5p, and miR-3622b-5p (Yang W. et al., 2016).

### GBAP1

GBA encodes lysosomal glucocerebrosidase and its mutations are associated with Parkinson’s disease. Recently, Tang et al. (2017b) demonstrated that GBAP1, a pseudogene of GBA, acted as a ceRNA for GBA by competing with miR-22-3p.

### INTS6P1

INTS6P1, a tumor suppressive pseudogene-derived lncRNA, exerted its roles in HCC by competitively binding to oncogenic miR-17-5p and thus upregulating its cognate gene INTS6 (Peng et al., 2015). Lui et al. (2017) suggested that INTS6 suppressed growth of HCC through Wnt pathway. Taken together, INTS6P1-miR-17-5p-INTS6-Wnt signaling pathway may be a potential therapeutic target in treating HCC.

### MSTO2P1

MSTO2P1 functioned as an oncogenic molecule, including enhanced growth, colony formation, migration and invasion in cervical cancer as mentioned above (Li R.K. et al., 2017). The mechanistic investigation revealed that MSTO2P1 exhibited these effects by regulating expression of miR-335 which has been well documented as a potential suppressor of gastric cancer progression (Sandoval-Borquez et al., 2017).

### PDIA3P1

hMTR4, which promotes RNA degradation, could bind to PDIA3P1, and this interaction was disrupted by Dox treatment (Xie et al., 2019). miR-125a/b and miR-124 directly targeted TRAF6, however, PDIA3P1 bound to miR-125a/b and miR-124 and relieved their suppression on TRAF6, thereby causing activation of NF-kB pathway (Xie et al., 2019). The novel hMTR4-PDIA3P1-miR-125/miR-124-TRAF6 axis may play a key role in chemoresistance of HCC.

### RACGAP1P

Wang M.Y. et al. (2019) lately confirmed that pseudogene RACGAP1 is an indispensable molecule for the malignant progression of HCC cells, which was dependent on RACGAP1/Rho/ERK signaling axis.

### RP11-424C20.2

UHRF1 is a parental gene of pseudogene RP11-424C20.2, it is significantly related with immune infiltration (Yang J. et al., 2019). RP11-424C20.2, a ceRNA, could upregulate UHRF1 expression *via* sponging miR-378a-3p. This axis regulated immune escape of THYM and LIHC by PD-L1 and IFN-gamma-mediated CLTA-4 pathway.

### RP11-564D11.3

According to bioinformatic analysis, Song et al. (2019) found that high expression of RP11-564D11.3 was obviously associated with poor overall survival of multitype cancer patients, such as HCC, mesothelioma, kidney diseases, lung adenocarcinoma, paraganglioma, and pheochromocytoma. Furthermore, seven miRNAs binding with RP11-564D11.3 were predicted, including miR-200b-3p, miR-200a-3p, miR-429, miR-101-3p, miR-9-5p, miR-200c-3p, and miR-141-3p.

### RSU1P2

Pseudogene-derived LncRNA, RSU1P2, was founded to be a tumor-promoting molecule in cervical cancer by against let-7a, next regulated N-myc, IGF1R and EphA4 expression (Liu et al., 2017c).

### SEPW1P

A recent study reported that piRNA-36712 restrained proliferation, invasion, migration and paclitaxel and doxorubicin resistance of breast cancer, while SEPW1P decreased piRNA-36712 expression by regulating miRNA-7 and miRNA-324, then inhibited P53, p21 and *E*-cadherin, but upregulated Slug (Tan et al., 2019).

### SUMO1P3

Recent research found that the expression level of SUMO1P3 was significantly upregulated in non-small cell lung cancer cell lines and cancer tissues. Furthermore, SUMO1P3 promotes NSCLC cells invasion and migration by binding and regulating miR-136 (Zhang et al., 2019).

### WTAPP1

WTAPP1, a molecular decoy for miR-3120-5p, promoted cell migration, invasion and tube formation both *in vitro* and *in vivo* by increasing MMP1 expression and activating PI3K/Akt/mTOR signaling (Li et al., 2018).

## Potential Dysregulated Mechanisms of Pseudogene-Derived lncRNAs in Human Cancer

**FIGURE 2:**
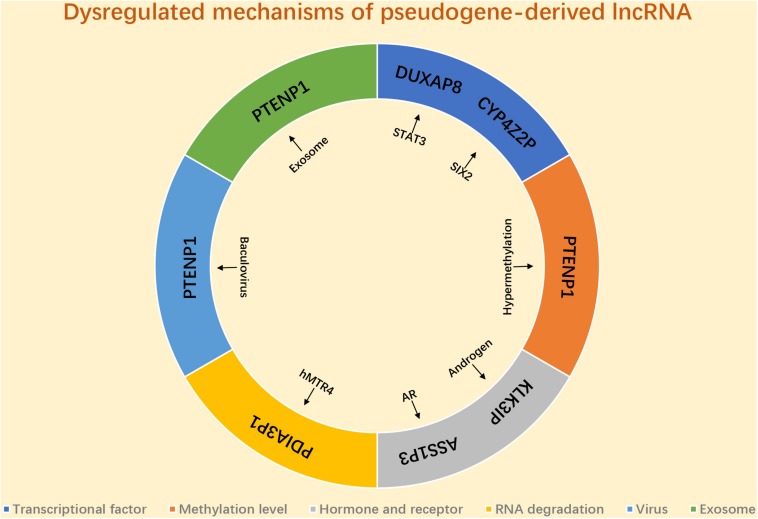
The potential mechanism responsible for dysregulation of pseudogene-derived lncRNA in cancer.

As we mentioned above, a variety of pseudogene-derived lncRNAs have been reported to be frequently dysregulated in various types of human cancer, and a lot attention has been paid to explore the function and action mechanisms of these identified pseudogene-derived lncRNAs in cancer. Only few potential dysregulated mechanisms of pseudogene-derived lncRNA have been reported (Figure 2). Several transcriptional factors are discovered to link to the mis-regulation of pseudogene-derived lncRNAs. For instance, Du et al. (2019) revealed that DUXAP8 upregulation in colorectal cancer was induced by STAT3; SIX2 played a positive role in CYP4Z2P expression in breast cancer (Zheng L. et al., 2019). Some studies demonstrated that the methylation level of pseudogenes linked to the alteration of pseudogene-derived lncRNAs’ expression (Kovalenko et al., 2013, 2018). Hormone and hormone receptor correlated with mis-regulation of pseudogene-derived lncRNAs. For example, Lu et al. (2006) suggested that KLK31P was stimulated by androgen in prostate cancer; the team of Wang K. et al. (2019) found that androgen receptor negatively modulated ASS1P3 expression in renal cell carcinoma. Emerging evidence showed that RNA stability-associated proteins participated in modulation of expression of pseudogene-derived lncRNAs. Human homolog of mRNA transport mutant 4 (hMTR4) led to PDIA3P1 degradation and caused its expression downregulation in HCC (Xie et al., 2019). A recent study confirmed that baculovirus mediated expression of PTENP1 in HCC (Chen et al., 2015). Besides, exosome also linked to the aberrant expression of pseudogene-derived lncRNA in cancer. Zheng et al. (2018) revealed that PTENP1, transmitted by exosome, significantly suppressed progression of bladder cancer. However, investigations into the mechanisms of pseudogene-derived lncRNAs’ upregulation or downregulation remain inadequate. More possible mechanisms causing deregulation of pseudogene-derived lncRNA in cancer need to be explored in the future.

## Conclusion and Future Perspectives

Pseudogenes are regarded as non-functional “Genomic evolutional junks” for a long time. During the past few years, researchers have reported that a variety of pseudogenes possess transcribed activities, and some of pseudogene-derived RNA transcripts are validated to play as key regulators in diverse biological processes (Wen et al., 2012). Expression and/or function dysregulation of these RNA transcripts account for the occurrence of multiple human disorders, containing cancer (Pink et al., 2011; Poliseno, 2012). Therefore, performing an all-round overview regarding the expression, function and molecular mechanisms of pseudogene-derived lncRNAs in human cancers may be helpful for developing effective anti-cancer measures.

Benefiting from the advancement of high-throughput sequencing technology and development of bioinformatics analysis, a growing number of pseudogene-derived lncRNAs have been identified, annotated and functionally predicted (Kalyana-Sundaram et al., 2012; Han et al., 2014). As reported, pseudogene-derived lncRNAs are frequently mis-regulated in diverse diseases. But, to date, the role of majority of pseudogene-derived lncRNAs remains unknown. Low expression abundance and high tissue specificity may hamper the interpretation of their functions. The beginning of functional studies for most of pseudogene-derived lncRNAs is usually the cognate genes of pseudogenes. The high homology of pseudogene-derived lncRNAs and transcripts from their cognate genes may be another drag force to impede investigation progression of pseudogene-derived lncRNAs’ functions. Of note, the influence of pseudogene-expressed transcripts needs to be excluded when researchers are performing the functional investigations for transcripts from cognate genes.

ceRNA hypothesis, proposed by Salmena et al. (2018) presents a new language among messenger RNAs, lncRNAs and circRNAs by using miRNA response elements. Pseudogene-derived lncRNA transcripts make up a part of lncRNAs. To the best of our knowledge, no studies regarding pseudogene-derived circRNAs have been reported although Dong et al. (2016) proposed that circRNAs can be retrotranscribed and finally inserted back into the host genome as processed pseudogenes. With the deepening of research, pseudogene-derived circRNA transcripts may be gradually discovered in the future.

To date, most of studies regarding molecular action mechanisms of pseudogene-derived lncRNAs mainly focused on the ceRNA hypothesis by competing shared miRNAs with cognate genes or non-cognate genes (Wei Y. et al., 2017; Wu et al., 2018). As a subtype of lncRNA, pseudogene-derived lncRNA may also exert its regulatory effects in cancer by other mechanisms, such as binding to transcription factors, producing miRNA/piRNA and encoding proteins (Bhan et al., 2017; Renganathan and Felley-Bosco, 2017). However, these fields are still inadequate. Moreover, most of individual transcripts can’t eventually compromise miRNA activity (Thomson and Dinger, 2016). In the future, more corresponding work should be performed. Of course, the current findings about roles and mechanisms of known pseudogene-derived lncRNAs need to be further precisely validated by basic lab experiments and large clinical trials. Despite all this, finding novel pseudogene-derived lncRNAs as well as circRNAs, identifying their roles in different cancer types and subtypes and developing these pseudogene-derived transcripts-based strategies for diagnosis, therapy and prognosis exhibit very promising in overcoming cancer.

## Author Contributions

WL and PF designed this work. WL collected the reference and drafted the manuscript. BD polished the language. PF critically revised the manuscript. All authors read and approved the final version of the manuscript.

## Conflict of Interest

The authors declare that the research was conducted in the absence of any commercial or financial relationships that could be construed as a potential conflict of interest.

## Abbreviations

ceRNA: competing endogenous RNA
HBV: hepatitis B virus
HCC: hepatocellular carcinoma
lncRNA: long non-coding RNA
miRNA: microRNA
MRE: miRNA response element
ncRNA: non-coding RNA.
